# Multi-omics Mendelian Randomization Reveals Immunometabolic Signatures of the Gut Microbiota in Optic Neuritis and the Potential Therapeutic Role of Vitamin B6

**DOI:** 10.1007/s12035-025-04923-4

**Published:** 2025-04-11

**Authors:** Jingzhi Wang, Xuehao Cui

**Affiliations:** 1 Department of Radiotherapy Oncology, The Affiliated Yancheng First Hospital of Nanjing University Medical School, The First People’s Hospital of Yancheng, Yancheng, Jiangsu, Yancheng No.1 People’s Hospital, Nanjing University, Yancheng, China; 2 John Van Geest Centre for Brain Repair and MRC Mitochondrial Biology Unit, Department of Clinical Neurosciences, University of Cambridge, Cambridge, CB2 0PY UK; 3 Cambridge Eye Unit, Addenbrooke’S Hospital, Cambridge University Hospitals, Cambridge, UK

**Keywords:** Optic neuritis, Mendelian randomization, Immune cells, Gut microbiota, Vitamin B6

## Abstract

Optic neuritis (ON) is an inflammatory condition often linked to autoimmune disorders like multiple sclerosis (MS) and neuromyelitis optica spectrum disorder (NMOSD). Despite its low incidence, ON can lead to severe, irreversible visual impairment. Emerging evidence suggests the gut microbiota (GM) may influence ON through complex immune and metabolic pathways, yet the mechanisms remain underexplored. This study employed a multi-omics Mendelian randomization (MR) approach to assess the causal relationships between GM taxa and ON. We analyzed data from extensive genetic and metabolic profiling, examining the causal effect between 473 GM taxa, 731 immune cells/traits, 1400 metabolites, vitamin B6, and ON. Our methodology involved two-sample MR analysis to establish causality and mediation analysis to explore the mechanisms between GM and ON and potential therapeutic strategies. We identified 27 GM taxa with a direct causal relationship to ON risk. Further analysis highlighted three GM taxa with particularly strong associations. Mediation analysis revealed specific metabolic and immune pathways through which these taxa exert their effects, including notable interactions with vitamin B6 metabolism, which appeared to mediate some of these relationships. We found that vitamin B6 could be a potential therapeutic method by acting on GM in ON. This study advances our understanding of the complex etiology of ON and highlights the potential of GM as a target for therapeutic strategies. The significant role of vitamin B6 suggests new avenues for prevention and treatment, emphasizing the need for further research into diet-based interventions.

**Supplementary Information:**

The online version contains supplementary material available at 10.1007/s12035-025-04923-4.

## Introduction

Optic neuritis (ON) is an inflammatory optic neuropathy frequently associated with autoimmune neurological disorders such as multiple sclerosis (MS), myelin oligodendrocyte glycoprotein antibody-associated disease, and neuromyelitis optica spectrum disorder (NMOSD) [1]. Despite ON affecting only about 2.18 individuals per 100,000/year [2], severe immune-mediated attacks can rapidly result in blindness and paralysis if the condition remains undiagnosed and untreated [3]. NMOSD is a chronic inflammatory autoimmune disease of the central nervous system (CNS), characterized by severe visual impairments, movement disorders, and potentially fatal outcomes, affecting 0.5 to 4 individuals per 100,000 with a female-to-male ratio of 9:1 [4–6]. MS is a chronic inflammatory and degenerative CNS disease affecting nearly three million worldwide, with pathology driven by neuroimmune interactions [7]. Although it is well-known that ON is commonly linked to NMOSD and MS, the specific mechanisms underlying ON remain poorly understood [8]. ON often leads to severe, irreversible vision damage, with symptoms such as vision loss and eye pain that can deteriorate quickly, and recovery that may extend beyond a year, emphasizing the urgent need to enhance our understanding of ON’s mechanisms and to develop effective preventive and therapeutic strategies in clinical practice [9].

The gut microbiota (GM) is closely associated with the development of inflammatory, metabolic, mental, and immune disorders, in addition to affecting neurotransmitter functions [10–15]. The GM influences brain function and neurodevelopment via the microbiota-gut-brain axis by regulating physiological processes through three interconnected pathways: immune, neuronal, and endocrine/systemic [16]. Recent studies have confirmed that the GM influences immune diseases such as ON [17–20]. Some researchers have discovered that inflammation stemming from an ecological imbalance in the GM can lead to NMOSD, causing damage to the spinal cord and optic nerve [21]. A study revealed that GM differs significantly between patients with demyelinating optic neuritis (DON) and healthy controls, with treatments like intravenous methylprednisolone pulse potentially restoring its diversity and implicating its role in DON pathogenesis [22]. Previous studies indicated that GM and their hosts exhibit a complex trans-kingdom symbiosis, dynamically regulating immune, metabolic, and nervous system functions through the “gut-brain axis,” with imbalances in the GM linked to various neurological disorders [23]. Similarly, researchers have described the “gut-brain” axis as a bidirectional communication mechanism between the CNS and the GM, which allows targeted gut microbiota therapy to have a certain effect on MS [24]. Additionally, recent research has established a connection between the GM and age-related macular degeneration (AMD) through what is known as the gut-retina axis [25–27]. Given that the optic nerve serves as an extension from the brain to the eye, the GM could potentially influence conditions like ON or retinal damage. Although previous studies had suggested a potential causal relationship between GM and ON [9], no research has yet detailed the pathways through which GM may influence ON, nor has any study explored the role of GM in the treatment of ON.

Unlike randomized controlled trials (RCT), Mendelian randomization (MR) studies use genetic variations as proxies for exposure to mitigate the effects of confounding factors, such as diet, and reverse causality when assessing causal relationships with outcomes [28]. Studies employing MR analysis to examine the causal links between GM and neuroinflammatory diseases have become more refined. However, no studies have yet utilized MR and mediation MR analysis to investigate the specific immune mechanisms and metabolic pathways between GM and ON or to examine the potential role of GM in its treatment. Therefore, this study evaluates the potential causal relationships between each GM taxa and ON through MR, further investigating how GM can regulate the risk of ON through various immune cells and metabolites. Additionally, the study explores which microelements can act on GM to play a positive role in the treatment of ON. This research deepens our understanding of the causal relationship between the GM and ON, and the pathogenesis of ON, providing new insights for the prevention and treatment of ON.

## Methods

### Study Design

**Fig. 1:**
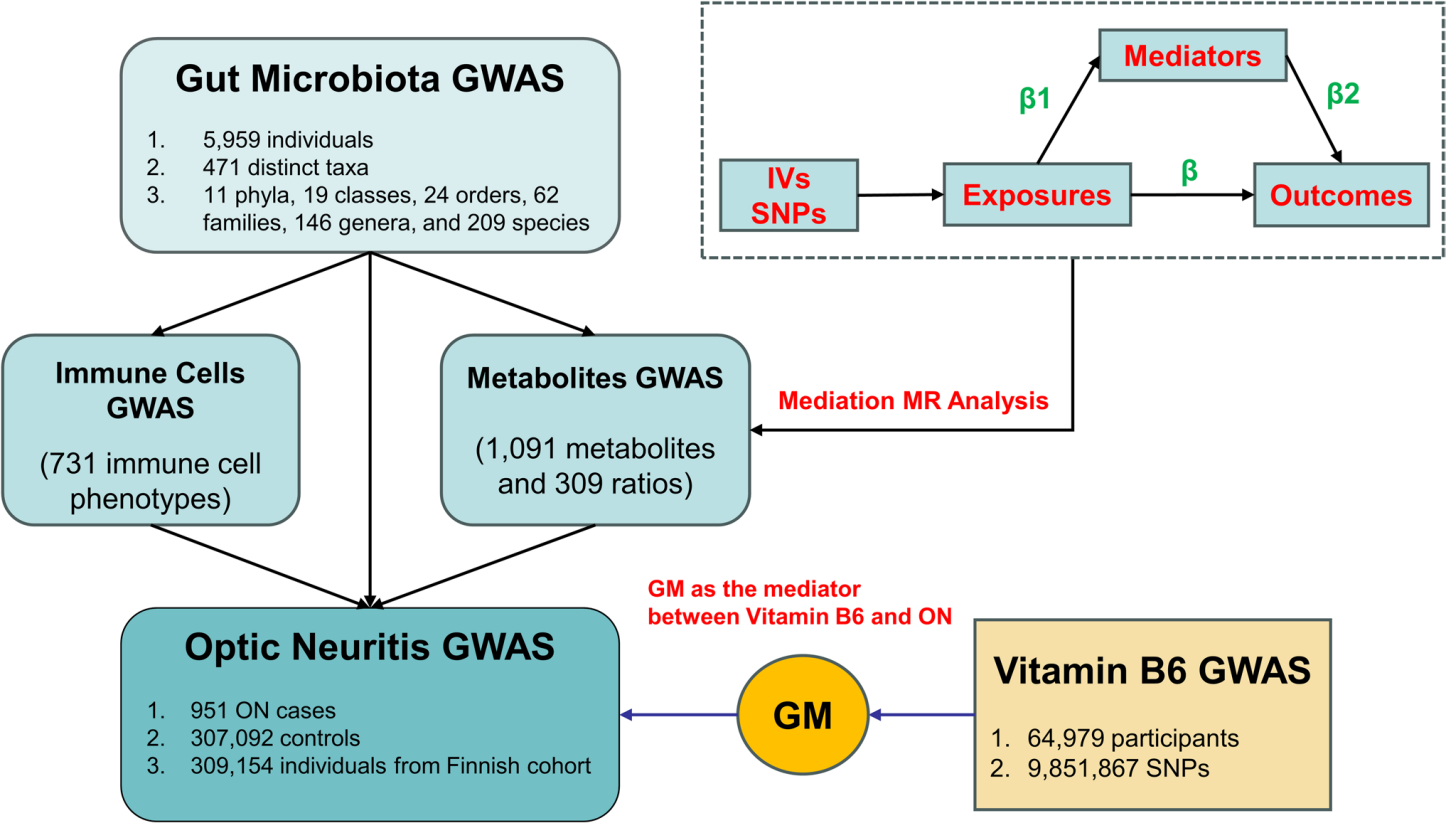
The study design and overview

Our study consists of three steps (Fig. 1). In the first step, we conducted a two-sample MR analysis to assess the causal relationships between 473 GM taxa and ON, as well as 731 immune cells/traits and 1400 metabolites/traits with ON. In the second step, we investigated the mediating effects of immune cells and metabolites on the causal relationship between GM and ON and performed enrichment analysis to explore the immune-metabolic mechanisms. In the third step, based on the MR analysis results from the previous steps, we explored the causal relationship between vitamin B6 and ON, revealing the potential therapeutic role of vitamin B6 in ON and the mediating effect of GM (Table S1).

To ensure the validity of the MR analysis, three assumptions had to be satisfied: (1) The genetic variants utilized in the analysis should demonstrate a significant association with the exposure; (2) the genetic variants chosen as instrumental variables (IVs) for exposure should be uncorrelated with confounding factors associated with both the exposure and outcome; and (3) there should be no horizontal pleiotropy, indicating that IVs can solely influence the outcome through exposures [29].

The study proceeded with two-step MR analyses to evaluate and quantify the mediating effects of selected mediators on the causal pathway between exposures and outcomes. The initial step involved using MR analysis to determine the causal impact (β1) of exposures on each mediator. Following this, the second step utilized MR analysis to assess the causal influence (β2) of each mediator on the risk of outcome. The proportion of each mediator was calculated by dividing the product of the mediation effect (β1 × β2) by the total effect between exposures and outcomes [30].

### Ethics Statement

This study utilized deidentified public summary-level data, freely available for download, to investigate the relationship between GM taxa, immune cells, metabolites, vitamin B6, and ON. The genome-wide association studies (GWASs) employed in this research were all approved by their respective institutional ethics committees.

### Exposure Sources of GM Taxa, Immune Cells, Metabolites, and Vitamin B6

The summary statistics for GM taxa were provided by the GWAS study from 5959 individuals enrolled in the FR02 cohort [31]. Using a genome-wide significance threshold (*P* < 5.0 × 10^−8^), we identified 473 distinct taxa from the Genome Taxonomy Database (GTDB), representing 17% of all tested taxa. This group included 11 phyla, 19 classes, 24 orders, 62 families, 146 genera, and 209 species associated with at least one genetic variant.

The GWAS data of immune cells/traits was obtained from a study aimed at exploring the genetic basis of immune cells/traits [32]. This research involved analyzing a wide range of genetic variations to find those related to immune cell characteristics, thereby gaining insights into their effects on immune system functionality. The study conducted 539 independent analyses to identify genetic variants linked to immune cell traits and assessed their functionality and impact. Using flow cytometry, 731 immune cell phenotypes were classified into four main groups: absolute cell counts (AC), median fluorescence intensity (MFI) indicating surface antigen levels, morphological parameters (MP), and relative cell counts (RC). The research specifically examined seven types of immune cells, T cells, B cells, dendritic cells (DCs), monocytes, other myeloid cells, natural killer cells, and Treg cells, aiming to understand the genetic factors influencing immune cell functionality.

The GWAS dataset on metabolites was sourced from a study focusing on the genetic determinants of metabolite profiles [33]. In this analysis, researchers examined 1091 metabolites and 309 ratios, identifying genetic associations for 690 metabolites and 143 ratios across a wide array of loci. Key genes that impact these metabolites were pinpointed, and through MR, the study revealed that specific metabolites and ratios causally affect diverse health traits and diseases. A significant finding was the association of elevated orotate levels with a higher risk of hip fractures, highlighting potential therapeutic targets grounded in the genetic interplay between metabolites and their links to diseases.

The GWAS data on vitamin B6 was derived from a substantial cohort of European ancestry within the UK Biobank (UKB). This dataset, compiled in 2018, included 64,979 participants and featured a comprehensive analysis of 9,851,867 SNPs. This large-scale genetic evaluation provides a robust foundation for exploring the genetic determinants associated with vitamin B6 levels.

### Outcome Source of Optic Neuritis

ON data were sourced from the FinnGen consortium’s seventh release (R7, 2022) (https://r7.finngen.fi/). This comprehensive dataset encompasses 16,962,023 variables collected from Finnish biobanks, alongside digital health record data from 309,154 individuals obtained from Finnish health registries. The diagnosis of ON was standardized according to the International Classification of Diseases, 10 th edition (ICD- 10) criteria. For the analysis, the data were rigorously adjusted for age, sex, genetic relatedness, genotyping batch, and the first ten principal components (PCs). This resulted in a final dataset comprising 951 ON cases and 307,092 controls, all of Finnish descent, for the genetic analysis of ON.

### Selection of Genetic Variants and Variants Harmonization

To adhere to the three fundamental assumptions of MR and ensure the accuracy of our findings, we conducted thorough quality checks on all single nucleotide polymorphisms (SNPs). We ensured that the SNPs selected were significantly associated with the exposure, with all SNPs linked to GM taxa, immune cells, and metabolites meeting the genome-wide significance threshold (*P* < 5 × 10^−8^). Additionally, we chose a separate set of SNPs below the locus-wide significance level (1 × 10^−5^) as instrumental variables to support robust conclusions. Linkage disequilibrium (LD) analysis was performed with strict criteria (R2 < 0.001, clumping distance = 10,000 kb) to align with MR assumptions. This step was executed using the *gwwasvcf* package in R [34]. The strength of the instruments was quantified by the *F*-statistic [35], while the variance explained was determined using the *r* [2, 36]. We harmonized genetic variants by aligning the effect sizes (betas) of different studies to the same effect allele using the *TwoSampleMR* package [37]. For cases where a specific exposure SNP was absent in the outcome dataset, we employed proxy SNPs with an *r* [2] > 0.8. The linkage disequilibrium (LD) matrix from the 1000 Genomes Project, specifically the European sample of Utah residents from North and West Europe, was used in our analyses. To minimize weak instrument bias and enhance the robustness of the MR analyses, we only retained results that were based on at least three independent shared SNPs with a mean *F*-statistic > 10.

### MR Analysis

All statistical analyses were conducted using R software (Version 4.3.0). The *TwoSampleMR* R package facilitated the MR analysis, exploring the causal relationships between exposures and outcomes. A *P*-value of less than 0.05 was deemed statistically significant, indicating evidence of a potential causal effect [38]. We employed various analytical methods to validate the findings of our study, including inverse variance-weighted (IVW) analysis, the weighted median (WM), the weighted mode, the simple mode, and the MR-Egger test. To estimate the overall impact of exposures on ON, the IVW method was used in the analysis. We chose either a fixed- or random-effects model for the IVW analysis based on the presence of heterogeneity. In cases where significant heterogeneity (*P* < 0.05) was detected, a random-effects IVW model was utilized. The beta, OR, and 95% confidence interval (CI) showed the effect size.

### Sensitivity Analysis

Sensitivity analyses were conducted to ensure the robustness of the MR findings regarding the causal relationship between exposures and ON. We utilized Cochrane’s Q method to assess heterogeneity within the IVs, with *P*-values less than 0.05 indicating potential heterogeneity. Pleiotropic effects were initially evaluated using the intercept from MR-Egger regression, considering *P*-values less than 0.05 as indicative of potential pleiotropy in the IVs. To further investigate pleiotropy, we applied the MR-PRESSO method using the “MR-PRESSO” package, which also allowed for the identification and exclusion of potential outliers. Additionally, the leave-one-out approach was implemented to exclude the influence of any single SNP, thus reinforcing the reliability of the MR analysis in determining the causality between exposures and ON [38].

### Mediation Analysis

The study proceeded with two-step MR to evaluate and quantify the mediating effects of selected mediators on the causal pathway between exposures and ON. The initial step involved using MR analysis to determine the causal impact (β1) of exposures on each mediator. Following this, the second step utilized MR analysis to assess the causal influence (β2) of each mediator on the risk of ON, factoring in adjustments for exposures. The contribution of each mediator to the overall effect was calculated by dividing the product of the mediation effect (β1 × β2) by the total effect. The standard errors for these mediation effects were computed employing the delta method [39].

### Enrichment Analysis About Metabolites

We performed enrichment analysis on metabolites with significant effects (*P* < 0.05) across ON, mapped against a curated reference of metabolic pathways. Enrichment ratios were calculated by dividing the count of metabolites within each pathway by the total count of metabolites cataloged in that pathway from the reference set. We employed the hypergeometric test to determine the statistical significance of enrichment for each pathway, which considers the size of both the reference set and the group-specific set.

## Result

### Association of Genetically Predicted GM and the Risk of ON

**Fig. 2:**
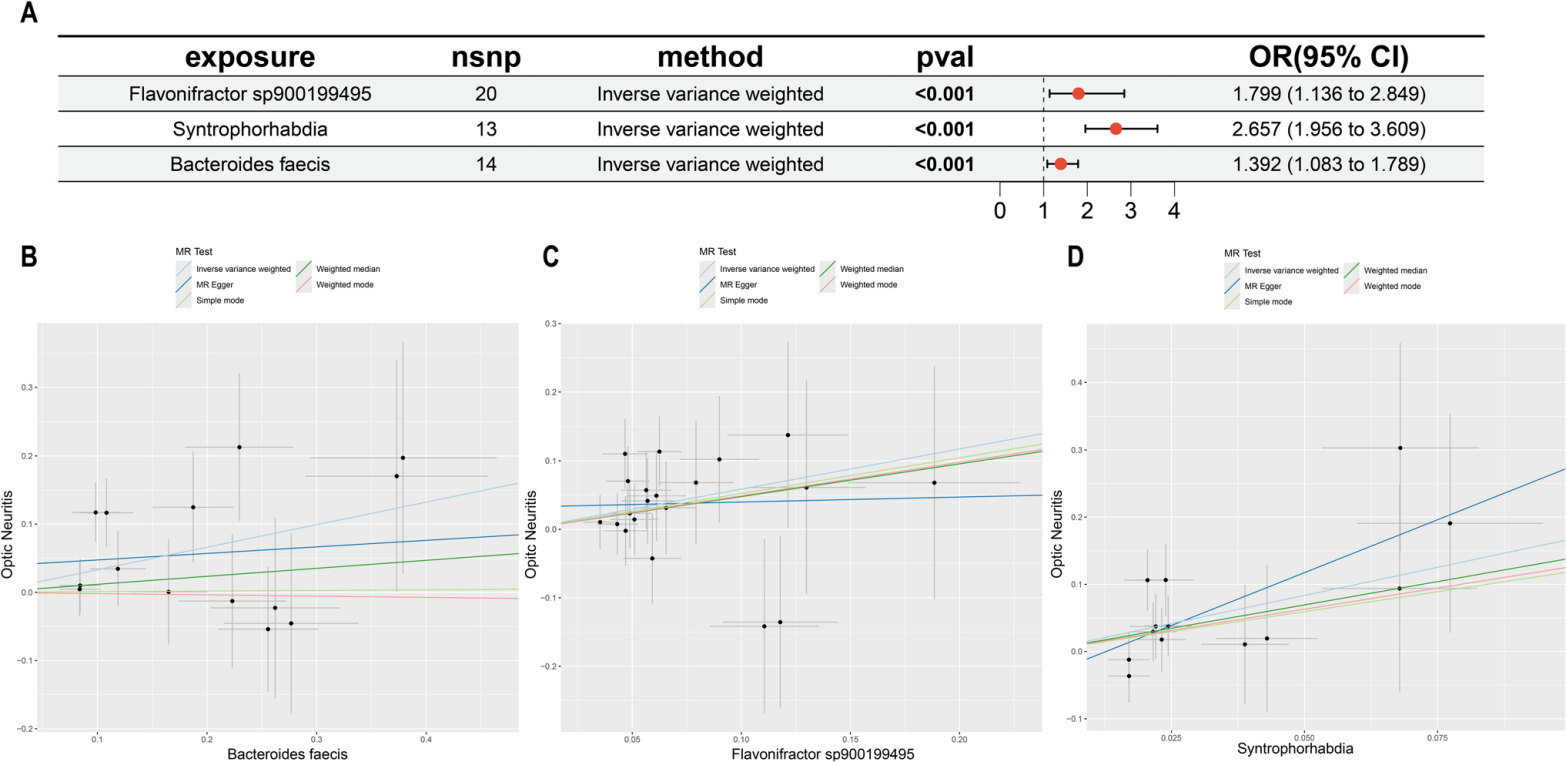
The MR result of GM and ON. **A** The result shows the causal effect between three GM taxa and ON. **B**–**D** The scatter plot of MR result of three GM taxa and ON

We first evaluated the relationships between the GM taxa and ON (Fig. 2). Using the IVW method, we identified 27 GM taxa with a causal relationship to ON (*P* < 0.05), and after validation using the Bonferroni method, the researchers found that three GM have an enhanced causal relationship with ON (*P* < 0.000106) (Table S2). The three GM with the most significant causal relationship to ON were as follows: Flavonifractor sp900199495 has a positive effect (OR = 1.799, 95% CI 1.136–2.849, *P* = 1.03E − 5), Syntrophorhabdia has a positive effect (OR = 2.657, 95% CI 1.956–3.609, *P* = 5.81E − 05), and *Bacteroides faecis* has a positive effect (OR = 1.392, 95% CI 1.083–1.789, *P* = 9.11E − 05). No heterogeneity or pleiotropy was detected in all three of these GM (Table S6).

### Causal Effect Between Immune Cells and the Risk of ON

**Fig. 3:**
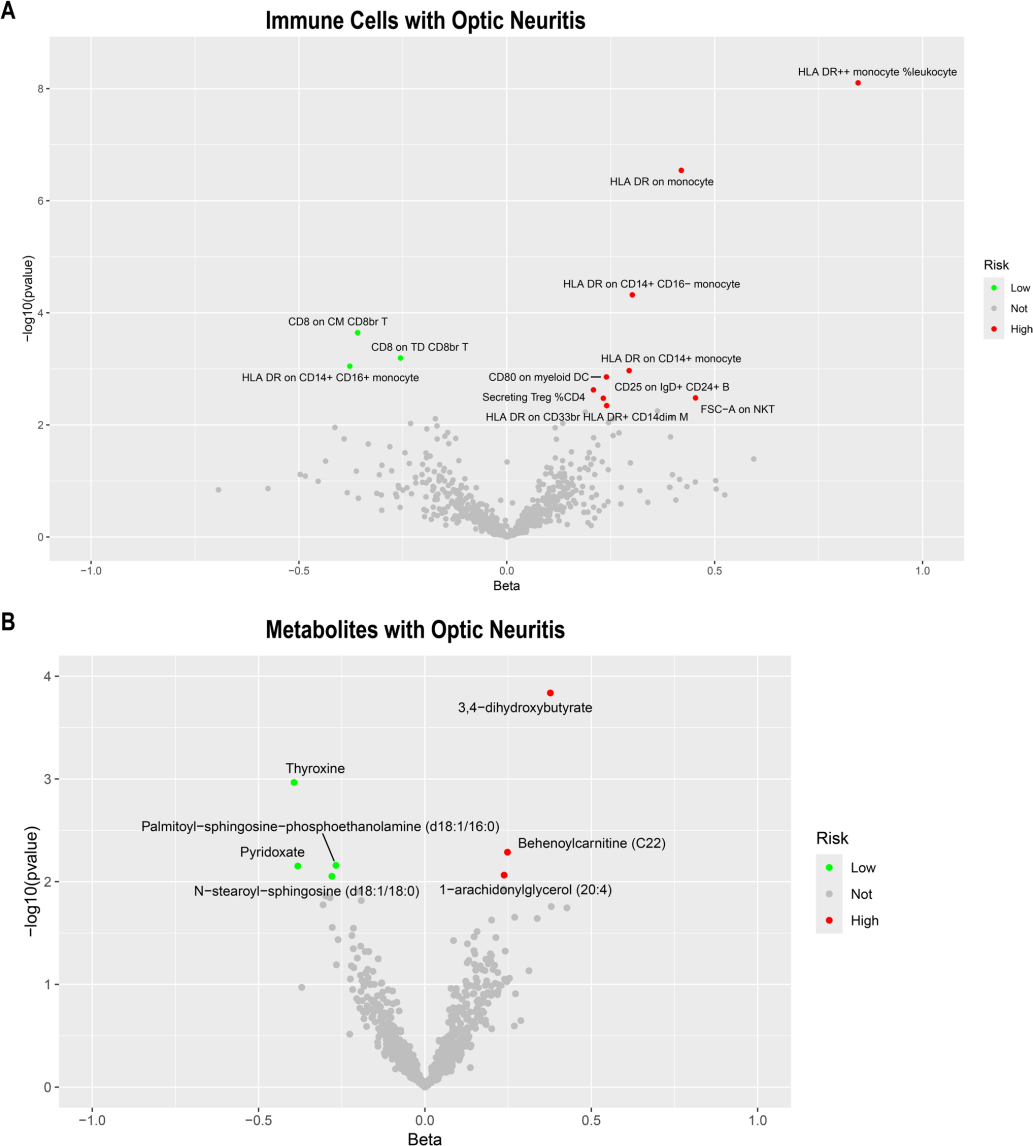
The volcano plot of the MR result. **A** The volcano plot shows the most significant immune cells that have a causal relationship with ON. **B** The volcano plot shows the most significant metabolites that have a causal relationship with ON

We then assess the causal effect between immune cells and ON (Fig. 3A). We found that 55 immune cells/traits have a causal relationship to ON (*P* < 0.05) (Table S3). We applied the Benjamini-Hochberg (BH) method to adjust our results, obtaining enhanced FDR-adjusted *P*-values (FDR-P). We found that four immune cells/traits had FDR-*P*-values less than 0.05, five had values less than 0.1, and nine had values less than 0.2. The five immune cells with the most significant causal relationship to ON were as follows: HLA DR +  + monocyte (beta = 0.845, FDR-*P* = 5.77E − 6), HLA DR on monocyte (beta = 0.420, FDR-*P* = 0.0001), HLA DR on CD14 + CD16-monocyte (beta = 0.302, FDR-*P* = 0.0116), CD8 on CM CD8br T cell (beta =  − 0.359, FDR-*P* = 0.0412), and CD8 on TD CD8br T cell (beta =  − 0.256, *P* = 0.0936). Additionally, among the 55 immune cells, the researchers can also see that there are 32 immune cells that have a positive causal relationship with ON, suggesting that these cells may increase the risk of ON. There are 23 immune cells with a negative causal relationship, suggesting that these cells may inhibit the occurrence of ON. This has a guiding significance for the future prevention and treatment of ON. No heterogeneity or pleiotropy was detected in all of these immune cells.

### Causal Effect Between Metabolites and the Risk of ON and Enrichment Analysis

We further evaluated the causal link between metabolites and ON, as depicted in Fig. 3B. This analysis revealed 34 metabolites demonstrating a causal relationship with ON at a significance level of *P* < 0.05 (Table S4). Among these, 16 metabolites were found to have a positive causal association, indicating a potential to elevate the risk of developing ON. Conversely, 18 metabolites exhibited a negative causal relationship, implying their role in reducing the likelihood of ON occurrence.

**Fig. 4:**
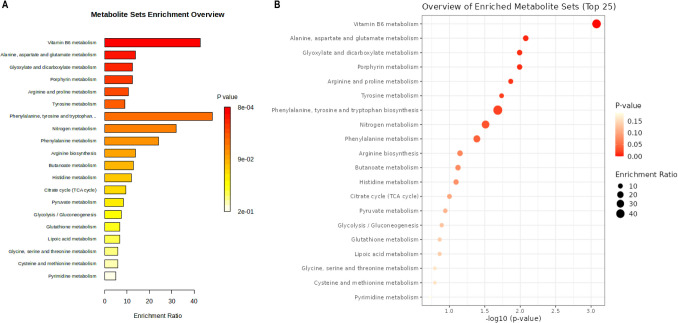
The enrichment analysis. **A** The metabolite enrichment of ON in different enrichment ratios. **B** The top 25 metabolic pathways associated with ON, with red being the most significant

We then conducted enrichment analysis on metabolites to identify potential metabolic pathways (Fig. 4). The researchers discovered that vitamin B6 metabolism had the most significant effect, followed by pathways related to alanine, aspartate, and glutamate metabolism and glyoxylate and dicarboxylate metabolism, as well as metabolic pathways associated with phenylalanine, tyrosine, and tryptophan metabolism. This suggests that these metabolism-related pathways might have a strong causal relationship with ON. Additionally, this indicates that targets associated with these pathways could potentially serve as effective therapeutic targets for ON.

### Metabolites and Immune Cells Mediate the Causal Effect of GM with ON

We employed mediation MR analyses to investigate the potential mediating role of metabolites and immune cells in the association between GM and ON (Table 1). We discovered that *Bacteroides faecis* could affect ON by acting on 1-linoleoyl-gpc and 1-linoleoyl-GPE. The researchers observed that *Bacteroides faecis* (beta = 0.331) was associated with an increased total risk of ON. For direct effects, *Bacteroides faecis* was associated with a decreased risk of 1-linoleoyl-gpc (beta =  − 0.148) and 1-linoleoyl-GPE (beta =  − 0.134). In addition, 1-linoleoyl-gpc (beta =  − 0.219) and 1-linoleoyl-GPE (beta =  − 0.219) have exhibited a causal relationship with decreased risk of ON. The proportions of the causal effect of *Bacteroides faecis* with ON mediated by 1-linoleoyl-gpc and 1-linoleoyl-GPE were 9.80% and 8.02% respectively. Besides, Flavonifractor sp900199495 could affect ON mediated by 3,4-dihydroxybutyrate and cortolone glucuronide. We found that Flavonifractor sp900199495 (beta = 0.587) was associated with an increased total risk of ON. For direct effects, Flavonifractor sp900199495 was associated with an increased risk of 3,4-dihydroxybutyrate (beta = 0.238) and cortolone glucuronide (beta = 0.309). In addition, 3,4-dihydroxybutyrate (beta = 0.378) and cortolone glucuronide (beta = 0.270) exhibited a causal relationship with an increased risk of ON. The proportions of the causal effect of *Bacteroides faecis* with ON mediated by 3,4-dihydroxybutyrate and cortolone glucuronide were 15.27% and 14.20% respectively. In addition, Syntrophorhabdia can affect ON by hyocholate. Syntrophorhabdia could have a decreased causal effect with hyocholate (beta =  − 0.511) and an increased risk of ON (beta = 1.672). Hyocholate had a decreased causal relationship with ON (beta =  − 0.511), and the proportion of the mediating effect of hyocholate between Syntrophorhabdia and ON was 9.35%.

**Table 1:** The mediation analysis between GM and ON mediated by immune cells and metabolites

Exposure	Mediator	Method	SNPs	*P*	β1	β2	β-total	Mediation ratio
*Bacteroides faecis*	1-Linoleoyl-gpc	IVW	14	0.0015	− 0.1478	− 0.2192	0.3306	9.80%
*Bacteroides faecis*	1-Linoleoyl-GPE	IVW	14	0.0043	− 0.1344	− 0.1973	0.3306	8.02%
Flavonifractor sp900199495	3,4-Dihydroxybutyrate	IVW	17	0.0068	0.2378	0.3770	0.5871	15.27%
Flavonifractor sp900199495	Cortolone glucuronide	IVW	17	0.0005	0.3089	0.2698	0.5871	14.20%
*Bacteroides faecis*	HLA DR + T cell	IVW	14	0.0472	− 0.1364	− 0.1945	0.3306	8.02%

In addition to metabolites, *Bacteroides faecis* can also influence ON by interacting with HLA DR + T cells. Our findings indicate that *Bacteroides faecis* has a significant impact on ON, with a positive association showing an increased total risk of ON (beta = 0.331). Conversely, for direct effects, *Bacteroides faecis* was associated with a reduced risk of influencing HLA DR + T cells (beta =  − 0.136). Furthermore, HLA DR + T cells have demonstrated a causal relationship with a decreased risk of ON (beta =  − 0.195). The proportion of the causal effect of *Bacteroides faecis* with ON, mediated by HLA DR + T cells, was quantified at 8.02%.

### The Potential Therapeutic Role of Vitamin B6 in ON and Mediating Effect of GM

**Fig. 5:**
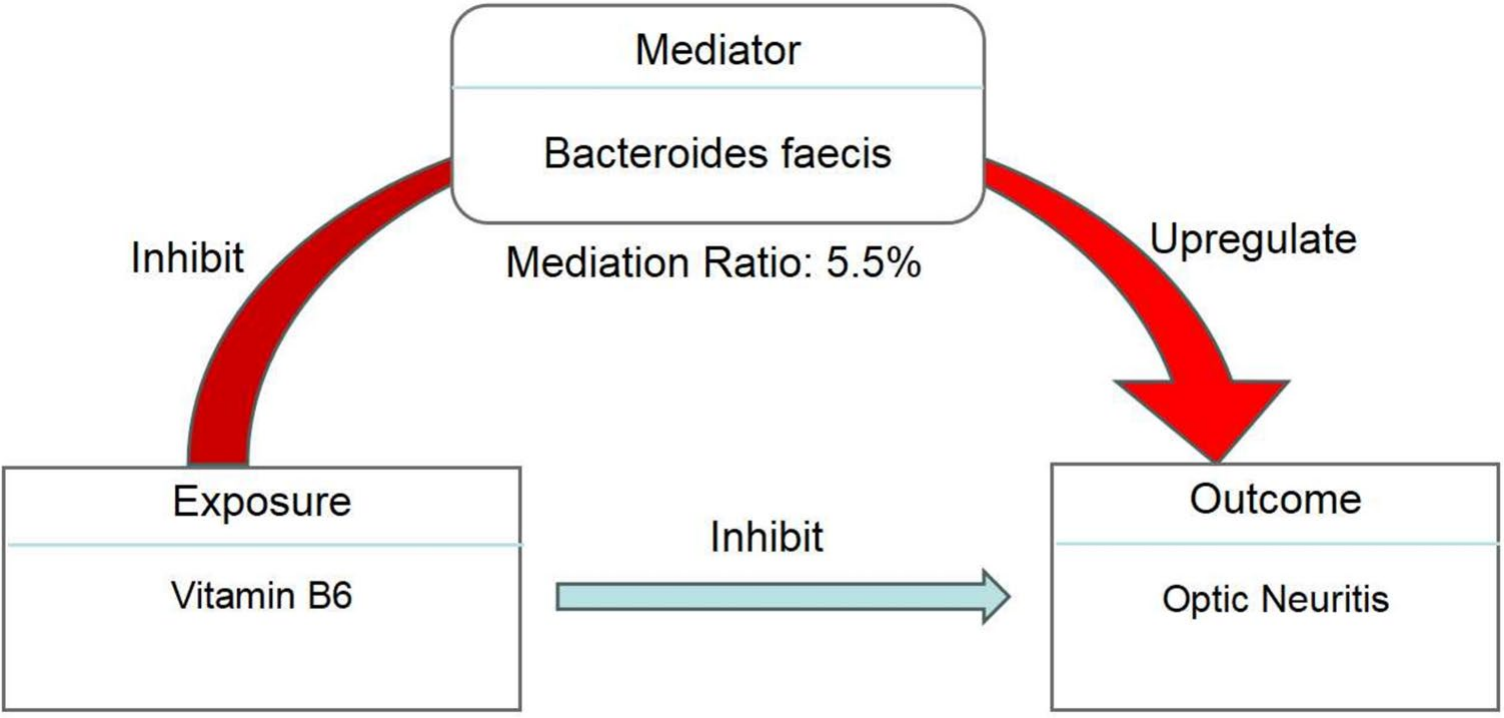
The mediation analysis of vitamin B6 and ON mediated by GM

In our previous metabolite enrichment analysis, we identified a significant causal relationship between the vitamin B6 metabolic pathway and ON. Consequently, we aimed to further investigate the relationship between vitamin B6 and ON. Using vitamin B6 as the exposure and ON as the outcome, we conducted a MR analysis and discovered a significant causal link between vitamin B6 and ON (OR = 0.38 95% CI 0.19–0.78, *P* = 0.008) (Table S5). The result suggested that vitamin B6 may have a protective or therapeutic effect with ON. Subsequently, we used vitamin B6 as the exposure factor to explore the mediating effect of the GM in the causal relationship between vitamin B6 and ON. We discovered a significant negative causal relationship between vitamin B6 and *Bacteroides faecis* (OR = 0.85, 95% CI 0.78–0.92, *P* = 7.02E − 5), while *Bacteroides faecis* may increase the risk of ON (OR = 1.39, 95% CI 1.08–1.79, *P* = 9.11E − 5) (Fig. 5). These findings suggest that vitamin B6 could exert a protective effect with ON by acting on *Bacteroides faecis*, with a mediating effect of 5.55%.

## Discussion

This study is the first to analyze the causal relationship between 473 gut microbiome taxa and ON, including the largest cohort based on the general population. Our study identifies Flavonifractor sp900199495, Syntrophorhabdus, and *Bacteroides faecis* as risk factors for ON. We found 55 immune cells/traits and 34 metabolites may have a causal relationship to ON. To further explore the potential roles of these three GM taxa in the pathology and treatment of ON, our mediation analysis revealed that Flavonifractor sp900199495 increases the risk of ON by promoting 3,4-dihydroxybutyrate and cortolone glucuronide. Conversely, *Bacteroides faecis* increases the likelihood of ON by inhibiting 1-linoleoyl-gpc, 1-linoleoyl-GPE, and HLA DR + T cells, which are protective against ON. Syntrophorhabdus, on the other hand, promotes the onset of ON by reducing hyocholate levels. In our metabolite enrichment analysis, we found that vitamin B6 appears to have a significant causal relationship with the incidence of ON. Consequently, we further investigated the causal relationship between vitamin B6, the GM, and ON. We discovered that vitamin B6 reduces the risk of ON and provides protection by inhibiting *Bacteroides faecis*. This suggests that vitamin B6 could be a potential therapeutic target for ON, and the GM may play a role in this process. These findings have important implications for identifying novel biomarkers in future investigations of ON and may inspire new prevention and therapeutic strategies for this condition.

The gut microbiota is composed of various microorganisms, including bacteria, viruses, fungi, and archaea, which reside in the human digestive tract. These microorganisms play a critical role in numerous physiological and metabolic functions, such as the digestion and absorption of nutrients, development of the immune system, and production of essential vitamins [40]. The GM’s composition changes with factors like age, diet, lifestyle, and location [41]. Imbalances in GM taxa can lead to diseases such as digestive, metabolic, autoimmune, and neurological disorders [42–44]. Recent studies have found a strong link between the gut microbiota and various neuropsychiatric and neurological disorders, including depression, anxiety, schizophrenia, autism spectrum disorders, Parkinson’s disease, migraine, and epilepsy [45]. GM interacts with their animal hosts through the gut-brain axis to influence the immune, metabolic, and nervous systems, impacting behaviors and potentially contributing to neurological disorders [23]. The retina is unique in its immune response, benefiting from multiple protective layers such as the blood-retina and blood-aqueous barriers, along with resistance and tolerance mechanisms [46]. Additionally, it relies on intrinsic defense mechanisms like microglia and the complement system to maintain normal function and prevent damage [47]. Recent studies have found a complex causal relationship between the gut microbiota and eye diseases, particularly those affecting the retina and optic nerve like diabetic retinopathy (DR), age-related macular degeneration (AMD), glaucoma, and ON [9, 25, 48–50]. The gut-retina axis represents a bidirectional communication pathway between the GM and the retina, implicating GM dysregulation in the development and progression of ocular diseases [51].

Immune diseases frequently result in ON, and while the link between specific GM taxa and immune conditions has been previously studied, it is speculated that changes in GM taxa abundance may prompt responses from the immune system [52]. A study detected an effect of increased abundance of *Bacteroides faecis* on attention-deficit hyperactivity disorder (ADHD) [53]. In a study about the gut-brain axis and its association with seven brain-related diseases (attention-deficit hyperactivity disorder, autism spectrum disorder, schizophrenia, Alzheimer’s disease, Parkinson’s disease, major depressive disorder, and bipolar disorder), it was found that the abundance of Flavonifractor is significantly higher in disease groups compared to healthy controls. This suggests that Flavonifractor may contribute to the disease process by promoting neuroinflammation and activating microglia, thereby increasing the risk of these disorders [54]. A study revealed Flavonifractor may potentially play a role in both Alzheimer’s disease and dysmetabolism, as Flavonifractor prevalence is inversely associated with health [55]. Another study found that the abundance of Bacteroides and Flavonifractor is significantly higher in the Parkinson’s disease population compared to the healthy control group, suggesting a significant association between these two GM and the gut-brain axis [56]. A study suggests that the ratio of Bacteroidetes to Firmicutes, dominant phyla in the gut microbiota, may be considered a biomarker associated with the development of DR [57]; another study revealed that Bacteroides were significantly and positively correlated with inflammatory factors in DR [58]. An animal experiment found that the abundance of Bacteroides significantly increased in mice with retinitis pigmentosa (RP) [59]. All of these studies suggested a complex association between the GM and neurological and ocular diseases. Furthermore, through mediation analysis, we have identified that these GM taxa may increase the risk of ON by modulating immune cells and metabolic pathways, providing a basis for further research into treatment methods for ON.

In the enrichment analysis of metabolites associated with ON identified through MR analysis, we found a significant correlation between vitamin B6-related metabolic pathways and ON. Vitamin B6 consists of a group of six water-soluble compounds, with pyridoxal phosphate being the active form. It acts as a cofactor for numerous reactions in the body and is found in poultry, fish, and plant-based foods [60]. Additionally, it plays a role in regulating homocysteine levels, which are associated with oxidative stress and apoptosis in retinal ganglion cells [61]. The relationship between vitamin B6 and neurological disorders is complex. Research indicates that both vitamin B6 deficiency and high B6 intake have been identified as risk factors for developing peripheral neuropathy [62]. A study found that vitamin B6 protected primate retinal neurons from ischemic injury, suggesting its potential protective role in the pathological process of optic nerve [63]. There exists a complex physiological relationship between vitamin B6 and the GM. Many GM species have the ability to produce vitamin B6, while the levels and activity of these GM species can also be regulated by the levels of vitamin B6 [64]. In our MR analysis, we found a significant negative causal relationship between vitamin B6 and ON, and it can exert a protective effect with ON by inhibiting Bacteroides, suggesting the potential application of vitamin B6 in the treatment of optic neuritis. With early and accurate diagnosis of ON, timely administration of high-dose corticosteroids, and, in some cases, plasmapheresis, the loss of high-contrast vision can be prevented, contrast sensitivity can be improved, and color vision and visual fields can be preserved [1]. According to our research findings, vitamin B6 may potentially become an effective therapeutic target for ON, but animal experiments and clinical trials are still needed to validate its therapeutic efficacy.

Several SNPs have been identified as key genetic regulators of vitamin B6 metabolism, particularly in genes such as ALPL, PDXK, and PNPO, which play crucial roles in the enzymatic conversion of vitamin B6 to its active form pyridoxal 5′-phosphate (PLP). For instance, rs4654748 in ALPL and rs2010795 in PDXK have been robustly associated with circulating PLP levels in large-scale GWAS analyses [65]. Although such SNPs could theoretically act as confounding variables in MR, our analysis incorporated rigorous sensitivity testing including MR-Egger and MR-PRESSO, which revealed no significant evidence of horizontal pleiotropy. These findings suggest that the causal relationship between genetically predicted vitamin B6 levels and ON is unlikely to be driven by the pleiotropic effects of B6-related SNPs. However, future studies may explore stratified MR using SNPs directly involved in B6 metabolism versus those reflecting transport or systemic regulation. Although we identified a significant protective effect of vitamin B6 on ON and highlighted its mediation through gut microbiota, we were unable to directly assess the relationship between genetically predicted vitamin B6 levels and neurological disability scores such as Expanded Disability Status Scale (EDSS) in MS patients due to the unavailability of public GWAS summary statistics on EDSS. Future studies incorporating clinical scores such as EDSS and vitamin B6 levels in MS cohorts are warranted to validate the translational significance of our findings.

## Study Strengths and Limitations

Our study has several significant strengths. Firstly, we conducted MR analysis using GWAS data containing 473 gut microbiota species for the first time to investigate the association between gut microbiota and ON, identifying three GM taxa that significantly increase the risk of ON. Secondly, we employed mediation analysis for the first time to explore potential immunometabolic mechanisms between GM and ON, revealing causal relationships between GM, certain metabolites, and immune cells. Thirdly, our analysis identified the potential protective role of vitamin B6 in ON, which was further validated through MR analysis, elucidating that vitamin B6 may reduce the risk of ON by acting on Bacteroides, suggesting its potential as a therapeutic target for ON.

This study is subject to several limitations. Firstly, the analysis was conducted using data from the European population, which may limit its generalizability. Secondly, despite efforts to identify and eliminate outlier variants, the potential for horizontal pleiotropy to affect the findings cannot be entirely ruled out. Thirdly, the genetic risk scoring method can also differentiate between GWAS of transgenic groups and GWAS of ON. However, to protect the privacy and genetic information of participants, we cannot perform calculations without obtaining individual-level data beforehand. Lastly, despite the significant correlation revealed in the causal relationship analysis between vitamin B6 and ON, the genetic prediction of vitamin B6 mediated by GM on the protective effect with ON in this study is 5.5%, indicating a relatively low influence. Consequently, further research is necessary to assess the impact of other mediators to enhance our understanding of the therapeutic efficacy of vitamin B6 in ON.

## Conclusion

In conclusion, this study is pioneering in thoroughly elucidating the causal links among GM taxa, immune cells, and metabolites with ON through MR, revealing key GM taxa, metabolic pathways, and immune-related mechanisms. The study also identifies the potential therapeutic role of vitamin B6 mediated by GM in ON. These findings are important for ON’s prevention and treatment, directly impacting patient care by offering novel insights into therapeutic strategies. The findings underscore the importance of focusing future research on the intricate gut-brain axis, gut-retina axis, and immune-metabolic mechanisms associated with ON, aiming to enhance patient outcomes through targeted interventions.

## Supplementary Information

Below is the link to the electronic supplementary material.

Supplementary file1 (XLS 25 KB)

Supplementary file2 (XLS 54 KB)

Supplementary file3 (XLS 174 KB)

Supplementary file4 (XLS 185 KB)

Supplementary file5 (XLS 23 KB)

Supplementary file6 (XLS 23 KB)

Supplementary file7 (XLS 32 KB)

Supplementary file8 (XLS 23 KB)

## Data Availability

No datasets were generated or analysed during the current study.
